# Human Trial for the Effect of Plasma-Activated Water Spray on Vaginal Cleaning in Patients with Bacterial Vaginosis

**DOI:** 10.3390/medsci10020033

**Published:** 2022-06-18

**Authors:** Yongwoo Jang, Junsoo Bok, Dong Keun Ahn, Chang-Koo Kim, Ju-Seop Kang

**Affiliations:** 1 Department of Pharmacology, College of Medicine, Hanyang University, Seoul 04736, Korea; 2 Department of Biomedical Engineering, Hanyang University, Seoul 04736, Korea; bokjs1401@hanyang.ac.kr; 3 Department of Media Communication, Hanyang University, Seoul 04736, Korea; kahn@hanyang.ac.kr; 4 Department of Chemical Engineering and Department of Energy Systems Research, Ajou University, Suwon 16499, Korea; changkoo@ajou.ac.kr

**Keywords:** plasma-activated water, underwater plasma discharge, bacterial vaginosis, vaginal cleaning

## Abstract

Underwater plasma discharge temporally produces several reactive radicals and/or free chlorine molecules in water, which is responsible for antimicrobial activity. Hence, it can simply sanitize tap water without disinfectant treatment. Additionally, the spraying technique using cleaning water exploits deep application in the narrow and curved vaginal tract of patients. Herein, we attempted a clinical trial to evaluate the vaginal cleaning effect of spraying plasma-activated water (PAW) to patients with vaginitis (46 patients). The efficacy was compared with treatment with betadine antiseptics used to treat bacterial vaginosis (40 patients). To evaluate the cleaning effect, Gram staining of the vaginal secretions was conducted before and after spraying PAW or betadine treatment (BT). Consequently, PAW-sprayed (PAWS) patients (22.3%) showed a better vaginal cleaning effect against Gram-positive and -negative bacteria than BT patients (14.4%). Moreover, 18 patients in the BT group showed worsened vaginal contamination, whereas five patients in the PAWS group showed worsened vaginal contamination. Taken together, the noncontact method of spraying cleaning water to the vagina exhibited a reliable vaginal cleaning effect without further bacterial infection compared with BT. Therefore, we suggest a clinical application of the spraying method using PAW for vaginal cleaning to patients with vaginitis without disinfectants and antibiotics.

## 1. Introduction

Bacterial vaginosis is the most common disease of vaginitis and is caused by unbalanced changes in the vaginal microbiome [1]. Its abnormal vaginal microbiota is likely associated with a reduced overall number of *Lactobacilli* species and the predominance of anaerobic microorganisms such as *Gardnerella vaginalis* and *Atopobium vaginae* species [2,3]. Generally, the clinical symptoms present vaginal discharges with a fish-like odor and urinary pain.

Currently, it is believed that the overgrowth of anaerobic species, predominantly *Gardnerella vaginalis*, creates a structured and polymicrobial biofilm barrier that is strongly attached to the vaginal epithelium [4,5]. In fact, several studies have supported that *Gardnerella vaginalis* is most of the bacterial composition in vitro biofilm formation models [3,6,7,8]. The microbiota is embedded in a polymeric matrix of extracellular nucleic acids, polysaccharides, and proteins [9,10]. Thus, it is likely that vaginal bacteria within biofilms are difficult to clearly eliminate using antibiotics, which is a common treatment for bacterial vaginosis [11]. This situation is considered to be correlated with high recurrence rates of bacterial vaginosis.

As an alternative therapeutic approach, spraying plasma-activated water (PAW) would be a promising way to destroy biofilms and then remove infected bacteria firmly attached to biofilms. It is known that, because the cold atmospheric-pressure plasma is discharged in water, PAW is disinfected by plasma exposure [12,13]. Therefore, PAW has potential antimicrobial activity within biofilms without causing bacterial resistance [14,15,16,17].

Generally, plasma irradiation at atmospheric pressure induces various ionized gases and free radicals that originate primarily from oxygen and nitrogen gases in the air and water [18,19,20]. In particular, oxygen-derived free radicals such as superoxide anions and hydroxyl ions are responsible for the antibacterial activity [21,22,23]. Additionally, many previous studies also support that underwater plasma exposure to chlorinated tap water can increase free residual chlorine molecules such as hypochlorous acid and hypochlorite ions, eliminating harmful microorganisms [24,25]. Because tap water can potentially be contaminated, depending on the surrounding environment, it must be sanitized for use as cleaning water. In this regard, plasma discharge in water would be a promising technology for easily and safely disinfecting tap water. In a previous preliminary study, we found a potential of PAW to patients with bacterial vaginosis (5 patients) [26]. However, there are still few studies related to the bacterial vaginosis of PAW when compared to the numerous studies showing the antibacterial effect of PAW. Considering this point, the vaginal cleaning effect of spraying sterilized water using PAW and the antibacterial effect of PAW to bacterial pathogens related to bacterial vaginosis can be considered.

In this study, we first attempted to clarify the cleaning effect of the spraying PAW compared with betadine treatment as control in vaginitis patients (control group; 40 patients, experimental group; 46 patients).

## 2. Materials and Methods

### 2.1. Ethical Consideration

This study was a single-institution, randomized, and comparative study performed at a Roen medical center. The study was approved by the Institutional Review Board of the Korea National Institute for Bioethics Policy (P01-202109-11-003).

### 2.2. Patient Characteristics and Trial Design

Among the patients who visited the hospital for suspected vaginitis, the gynecologists observed the color, smell, and viscosity of vaginal discharge to first select suspected patients with bacterial vaginosis. Finally, clinical trial participants were selected for the following conditions: (1) women who tested positive for 9 STD (sexually transmitted disease) polymerase chain reaction (PCR) tests; (2) those who had not undergone a hysterectomy; and (3) those who were willing to voluntarily participate in the clinical trial and comply with the clinical trial plan. Ninety-four vaginitis-suspected patients participated in this clinical study. They were randomly divided into the control group (47 patients) and the experimental group (47 patients). The patients in the control group were treated with a topical betadine, and the patients in the experimental group were treated by spraying plasma-activated water for 1 min (approximately 150~200 mL). Finally, the number of STD-positive patients was 40 in the control group and 46 in the experimental group.

### 2.3. Preparation of Plasma-Activated Water

PAW was prepared using an underwater plasma-generating device as previously reported [26]. Briefly, the atmospheric plasma was discharged for 10 min in a cleaning solution container with 3 L of tap water. Subsequently, the plasma-activated water in the cleaning solution container was sprayed on the patients of the experimental group using a spraying nozzle for 1 min.

### 2.4. Methods

To identify bacterial vaginosis, PCR analysis was conducted for the following STD-related bacteria: Gardnerella vaginalis, Mycoplasma hominis, Mycoplasma genitalium, Neisseria gonorrhoeae, Trichomonas vaginalis, Ureaplasma urealyticum, Ureaplasma parvum, Chlamydia trachomatis, and Treponema pallidum. These target transcripts were measured by using the commercial kit according to the manufacturer’s instruction (INFINA^TM^ STI 12, BIOWITHUS, Seoul, Korea). The Gram-positive test was performed using the vaginal secretion of all patients in the control and experimental groups before and after the application of betadine treatment or plasma-activated water spraying. Gram and PCR tests were conducted according to the institutional protocol of a Roen medical center (Seoul, Korea). The classification of bacteria was determined by the Gram stain (positive or negative) and morphologies (round shape, rod shape, or oval shape). According to these criteria, the vaginal bacteria were classified into the following five types according to the results of Gram staining as follows: Gram-positive cocci, Gram-positive bacilli, Gram-positive coccobacilli, Gram-negative bacilli, and Gram-negative coccobacilli. In addition, the grades were counted as the average number of each bacterium that was visible in the field when the microscopic field moved at random 10 times. In this regard, the samples were divided into four stages according to the number of bacteria visible as follows: rare (0~1), few (2~4), moderate (5~30), and heavy (over 30) stages.

### 2.5. Statistical Analyses

**Figure 1:**
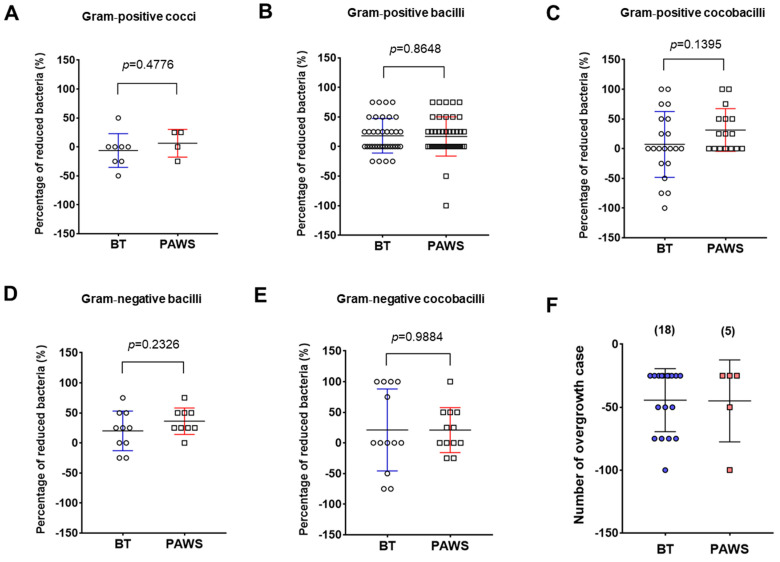
Beeswarm boxplot of the percentage of reduced bacteria, including Gram-positive cocci (**A**), Gram₋positive bacilli (**B**), Gram₋positive coccobacilli (**C**), Gram₋negative bacilli (**D**), and Gram₋negative coccobacilli (**E**), in betadine treatment (BT, blue) and plasma₋activated water sprayed (PAWS, red) patients. (**F**) Beeswarm boxplot of the number of overgrowth cases after the application of BT or PAWS. All the results are expressed as means ± standard deviation (SD).

The data in Figure 1 are expressed as means ± standard deviation (SD). Statistical significance was analyzed using Student’s t test. All statistical analyses were performed using GraphPad Prism 7 software (GraphPad Software Inc., San Diego, CA, USA). In Figure 1, all comparisons showed a non-significant difference between two groups (*p* >0.05).

## 3. Results

For this clinical study, we examined and analyzed the control (40 patients) and experimental (46 patients) groups, except for STD PCR-negative patients, from each group (Supplementary Tables S1 and S2). The control group was treated with the topical betadine, and the experimental group was treated by spraying plasma-activated water for 1 min. To determine a quantitative change in vaginal bacteria, we performed the Gram stain test from the vaginal secretion of all patients in the control and experimental groups before and after the application of betadine treatment or plasma-activated water spraying. The vaginal bacteria were classified into the following five types according to the results of Gram staining as follows: Gram-positive cocci, Gram-positive bacilli, Gram-positive coccobacilli, Gram-negative bacilli, and Gram-negative coccobacilli. When observing the results of Gram staining with a microscope, the samples were divided into four stages according to the number of bacteria visible as follows: rare (0~1), few (2~4), moderate (5~30), and heavy (over 30) stages. Based on this result, we evaluated the reduced number of bacteria before and after betadine treatment or plasma-activated water spraying according to the following standard. The 25% reduction in bacteria indicates a decrease in one stage, such as from heavy to moderate or from moderate to few. Thus, we deemed 50% for the decrease in two stages, 75% for the decrease in three stages, and 100% for the decrease in four stages.

Table 1 shows a reduced mean percentage of all bacteria, including Gram-positive and -negative bacteria. The PAWS (22.29%) patients had a slightly increased effect on vaginal cleaning compared with those with betadine treatment (BT, 14.37%). Specifically, the PAWS group showed a reduction of 20.56 ± 4.32 and 27.38 ± 6.88 in Gram-positive and -negative bacteria compared with the BT group (12.11 ± 5.04 and 20.65 ± 4.32), respectively.

Figure 1 is a beeswarm boxplot representing each value for a mean percentage of reduced bacteria between the BT and PAWS groups. The comparisons between BT and PAWS patients in the Gram-positive bacteria including cocci, bacilli, and coccobacilli are shown in Figure 1A–C, and their comparisons in the Gram-negative bacteria such as bacilli and coccobacilli are shown in Figure 1D,E. Overall, betadine swabs from patients with vaginitis show a broad treatment deviation in vaginal cleaning before and after treatment. In this regard, the PAWS group presented a better vaginal cleaning effect and narrow treatment deviation on vaginal cleaning compared with the BT group. Remarkably, the mean and standard deviation values for the reduced percentage of Gram-positive coccobacilli between the BT and PAWS groups were 7.14 ± 55.42 and 31.25 ± 35.94, respectively (Figure 1C). Regarding Gram-negative coccobacilli, the values were 21.15 ± 66.81 in the BT group and 20.83 ± 36.67 in the PAWS group (Figure 1E). Interestingly, 18 patients in the BT group showed worsened bacterial vaginosis, whereas 5 patients in the PAWS group showed worsened bacterial vaginosis (Figure 1F). Detailed information on the bacterial types and overgrowth is shown in Table 2. Additionally, Table 3 and Table 4 are raw results of patients in BT and PAWS groups for vaginal cleaning effect.

## 4. Discussion

In the present clinical study, we performed a randomized comparison of vaginal cleaning effects between BT and PAWS groups. As a result, we present a similar vaginal cleaning effect between BT (14.37 ± 44.07) and PAWS patients (22.29 ± 33.36) in the current study. For vaginal cleaning, current clinical results support that application with spraying PAW to patients is an effective method similar to BT. This human trials were designed as noninferiority trials, which determine whether a new experimental treatment is no less efficacious than an active control treatment already in use. If it is similar to the effect of betaine, and plasma-activated water spraying can replace the chemical betadine, it is believed that it has great advantages in treating patients with vaginitis. This finding is consistent with vaginal cleaning effect of PAW to patients (five patients) with bacterial vaginosis in a previous preliminary study [26]. In addition to the vaginal cleaning effect, the antibacterial effect of PAW on overgrown bacteria related to bacterial vaginosis could be expected, because numerous studies have shown the antibacterial effect of PAW [14,15,16,17].

Generally, swabs of betadine at the vaginal site of a suspected infection and the administration of antibiotics are common treatments for bacterial vaginosis. In the case of chemical antiseptic treatment, safety concerns are associated with topical use, particularly in the long term. Additionally, unexpected contamination is possible during the treatment process. Eighteen cases of bacterial vaginosis occurred in patients who received betadine treatment (Figure 1F). In some cases, bacterial infection is further exaggerated in the deep area of the vaginal tract that was not treated enough with betadine. The secondary contamination of treatment tools such as swabs can also occur.

Therefore, the PAW method, which is a topical non-contact method, is a safe, effective, and simple treatment method compared to the betadine swab method, which is a contact treatment method in the patient with bacterial vaginosis.

## 5. Conclusions

In this study, PAWS patients showed a slightly better outcome concerning vaginal cleaning with lower variability than those receiving BT. Considering these points, spraying cleaning water disinfected by plasma discharge to vaginitis patients is a promising cleaning method. 

## Figures and Tables

**Table 1:** A summary of the results of a percentage of reduced bacteria in the betadine-treated and plasma-activated water (PAW)-sprayed patients.

		BT	PAWS
All bacteria	Number of samples	87	83
	Average	14.37	22.29
	Standard deviation	44.07	33.36
	Standard error of mean	4.73	3.66
Gram-positive bacteria	Number of samples	64	62
	Average	12.11	20.56
	Standard deviation	40.33	34.03
	Standard error of mean	5.04	4.32
Gram-negative bacteria	Number of samples	23	21
	Average	20.65	27.38
	Standard deviation	53.65	31.53
	Standard error of mean	11.19	6.88

**Table 2:** A summary of the results of bacterial overgrowth case in the patients after treatment of betadine and PAW.

	Gram Stain	Before	After	Patient No.
Betadine-treated patients	Gram-positive cocci	-	few	B8
		-	rare	B17
		rare	few	B18
	Gram-positive bacilli	few	moderate	B2
		few	moderate	B7
		moderate	heavy	B14
		moderate	heavy	B27
	Gram-positive cocobacilli	few	moderate	B8
		rare	few	B17
		-	moderate	B25
		-	few	B33
		-	heavy	B38
		-	moderate	B39
	Gram-negative bacilli	-	rare	B2
		rare	few	B8
	Gram-negative cocobacilli	-	moderate	B5
		-	few	B14
		-	heavy	B17
PAW- sprayed patients	Gram-positive cocci	-	few	P5
	Gram-positive bacilli	-	heavy	P1
		-	few	P21
	Gram-negative cocobacilli	moderate	heavy	P14
		-	rare	P19

**Table 3:** A summary of the results of Gram stain in the betadine-treated patients. The minus (−) and plus (+) signs indicate the reduction and increase of bacteria, respectively.

Patient No.	Gram-Positive Cocci			Gram-Positive Bacilli			Gram-Positive Coccobacilli			Gram-Negative Bacilli			Gram-Negative Coccobacilli		
	Before	After	%	Before	After	%	Before	After	%	Before	After	%	Before	After	%
B1				few	rare	25				heavy	rare	75			
B2				few	moderate	−25				-	rare	−25			
B3				heavy	heavy	0									
B4				few	rare	25									
B5	few	few	0	few	few	0	heavy	moderate	25	moderate	few	50	-	moderate	−75
B6	few	-	50	few	rare	25	heavy	heavy	0				heavy	-	100
B7				few	moderate	−25	heavy	few	50						
B8	-	few	−50	moderate	moderate	0	few	moderate	−25	rare	few	−25			
B9				heavy	heavy	0									
B10				heavy	moderate	25									
B11				few	rare	25									
B12				heavy	few	50	heavy	-	100				heavy	-	100
B13				moderate	rare	50	moderate	-	75				heavy	-	100
B14				moderate	heavy	−25							-	moderate	−75
B15				moderate	moderate	0				few	-	50			
B16				heavy	rare	75	few	-	50	rare	rare	0			
B17	-	rare	−25	heavy	rare	75	rare	few	−25	rare	-	25	-	few	−50
B18	rare	few	−25	rare	-	25	heavy	heavy	0				heavy	heavy	0
B19				heavy	few	50									
B20				heavy	heavy	0	rare	-	25	rare	-	25			
B21	rare	rare	0	rare	rare	0	heavy	heavy	0	rare	-	25	few	few	0
B22				heavy	heavy	0									
B23	few	few	0	few	few	0	heavy	heavy	0				moderate	-	75
B24				heavy	moderate	25	heavy	heavy	0						
B25				heavy	rare	75	-	moderate	−75						
B26				rare	rare	0									
B27				moderate	heavy	−25									
B28				heavy	heavy	0									
B29	few	few	0	heavy	moderate	25	few	few	0	few	few	0	few	few	0
B30				heavy	rare	75									
B31				heavy	moderate	25									
B32				moderate	moderate	0									
B33				heavy	heavy	0	-	few	−50						
B34				heavy	heavy	0									
B35				moderate	few	25	heavy	-	100				heavy	-	100
B36				heavy	rare	75	moderate	-	75						
B37				few	-	50	heavy	heavy	0				moderate	moderate	0
B38				heavy	few	50	-	heavy	−100				-	heavy	−100
B39				heavy	heavy	0	-	moderate	−75						
B40				heavy	heavy	0									

**Table 4:** A summary of the results of Gram stain in the plasma-activated water sprayed patients. The minus (−) and plus (+) signs indicate the reduction and increase of bacteria, respectively.

Patient No.	Gram-Positive Cocci			Gram-Positive Bacilli			Gram-Positive Coccobacilli			Gram-Negative Bacilli			Gram-Negative Coccobacilli		
	Before	After	%	Before	After	%	Before	After	%	Before	After	%	Before	After	%
P1	heavy	moderate	25	-	heavy	−100	heavy	-	100	heavy	moderate	25	heavy	-	100
P2										heavy	rare	75			
P3										heavy	moderate	25			
P4										heavy	moderate	25			
P5	-	few	−50	moderate	moderate	0	moderate	moderate	0						
P6				heavy	heavy	0	few	-	50						
P7				few	rare	25	heavy	-	100						
P8				heavy	heavy	0									
P9				heavy	heavy	0									
P10				heavy	moderate	25									
P11				few	few	0									
P12				moderate	few	25	moderate	few	25				few	-	50
P13				heavy	rare	75							rare	rare	0
P14				moderate	moderate	0	heavy	heavy	0				moderate	heavy	−25
P15				few	few	0									
P16				heavy	moderate	25	heavy	heavy	0				heavy	heavy	0
P17				heavy	heavy	0									
P18				moderate	few	25									
P19				heavy	few	50	moderate	moderate	0				-	rare	−25
P20	rare	rare	0	rare	rare	0	heavy	heavy	0	rare	rare	0	few	few	0
P21				-	few	−50	heavy	few	50				heavy	few	50
P22				heavy	heavy	0	Few	rare	25						
P23				heavy	rare	75									
P24				heavy	heavy	0									
P25				heavy	rare	75	heavy	few	50	few	-	50			
P26				heavy	heavy	0									
P27				heavy	heavy	0									
P28				heavy	heavy	0									
P29				heavy	heavy	0									
P30				few	rare	25									
P31				heavy	heavy	0	few	few	0	rare	-	25	rare	-	25
P32				heavy	heavy	0									
P33				heavy	moderate	25									
P34				few	-	50	heavy	rare	75				moderate	moderate	0
P35				heavy	moderate	25									
P36				heavy	few	50									
P37				heavy	heavy	0									
P38				heavy	rare	75									
P39	few	rare	25				heavy	heavy	0	few	-	50	heavy	moderate	25
P40				heavy	moderate	25									
P41				heavy	moderate	25				few	-	50	few	-	50
P42				heavy	rare	75									
P43				heavy	few	50									
P44				heavy	moderate	25	rare	-	25						
P45				heavy	few	50									
P46				rare	rare	0									

## Data Availability

The data generated are included within the manuscript.

## Supplementary Materials

The following supporting information can be downloaded at: https://www.mdpi.com/article/10.3390/medsci10020033/s1, Table S1: A summary on the results of STD PCR test in the betadine-treated patients; Table S2: A summary on the results of STD PCR test in the plasma-activated water sprayed patients.

## References

[1] Russo R., Karadja E., De Seta F. (2019). Evidence-based mixture containing Lactobacillus strains and lactoferrin to prevent recurrent bacterial vaginosis: A double blind, placebo controlled, randomised clinical trial. Benef. Microbes.

[2] Verhelst R., Verstraelen H., Claeys G., Verschraegen G., Delanghe J., Van Simaey L., De Ganck C., Temmerman M., Vaneechoutte M. (2004). Cloning of 16S rRNA genes amplified from normal and disturbed vaginal microflora suggests a strong association between Atopobium vaginae, Gardnerella vaginalis and bacterial vaginosis. BMC Microbiol..

[3] Castro J., Machado D., Cerca N. (2019). Unveiling the role of Gardnerella vaginalis in polymicrobial Bacterial Vaginosis biofilms: The impact of other vaginal pathogens living as neighbors. ISME J..

[4] Swidsinski A., Mendling W., Loening-Baucke V., Ladhoff A., Swidsinski S., Hale L.P., Lochs H. (2005). Adherent biofilms in bacterial vaginosis. Obstet. Gynecol..

[5] Verstraelen H., Swidsinski A. (2019). The biofilm in bacterial vaginosis: Implications for epidemiology, diagnosis and treatment: 2018 update. Curr. Opin. Infect. Dis..

[6] Rosca A.S., Castro J., Franca A., Vaneechoutte M., Cerca N. (2021). Gardnerella Vaginalis Dominates Multi-Species Biofilms in both Pre-Conditioned and Competitive In Vitro Biofilm Formation Models. Microb. Ecol..

[7] Machado A., Cerca N. (2015). Influence of Biofilm Formation by Gardnerella vaginalis and Other Anaerobes on Bacterial Vaginosis. J. Infect. Dis..

[8] Gilbert N.M., Lewis W.G., Lewis A.L. (2013). Clinical features of bacterial vaginosis in a murine model of vaginal infection with Gardnerella vaginalis. PLoS ONE.

[9] Campoccia D., Montanaro L., Arciola C.R. (2021). Extracellular DNA (eDNA). A Major Ubiquitous Element of the Bacterial Biofilm Architecture. Int. J. Mol. Sci..

[10] Hoiby N., Ciofu O., Johansen H.K., Song Z.J., Moser C., Jensen P.O., Molin S., Givskov M., Tolker-Nielsen T., Bjarnsholt T. (2011). The clinical impact of bacterial biofilms. Int. J. Oral. Sci..

[11] Machado D., Castro J., Palmeira-de-Oliveira A., Martinez-de-Oliveira J., Cerca N. (2015). Bacterial Vaginosis Biofilms: Challenges to Current Therapies and Emerging Solutions. Front. Microbiol..

[12] Zhao Y.M., Ojha S., Burgess C.M., Sun D.W., Tiwari B.K. (2020). Inactivation efficacy and mechanisms of plasma activated water on bacteria in planktonic state. J. Appl. Microbiol..

[13] Zhou R.W., Zhou R.S., Prasad K., Fang Z., Speight R., Bazaka K., Ostrikov K. (2018). Cold atmospheric plasma activated water as a prospective disinfectant: The crucial role of peroxynitrite. Green Chem..

[14] Mai-Prochnow A., Zhou R., Zhang T., Ostrikov K.K., Mugunthan S., Rice S.A., Cullen P.J. (2021). Interactions of plasma-activated water with biofilms: Inactivation, dispersal effects and mechanisms of action. NPJ Biofilm. Microbiomes.

[15] Tan J., Karwe M.V. (2021). Inactivation and removal of Enterobacter aerogenes biofilm in a model piping system using plasma-activated water (PAW). Innov. Food Sci. Emerg. Technol..

[16] Zhou R., Zhou R., Wang P., Luan B., Zhang X., Fang Z., Xian Y., Lu X., Ostrikov K.K., Bazaka K. (2019). Microplasma Bubbles: Reactive Vehicles for Biofilm Dispersal. ACS Appl. Mater. Interfaces.

[17] Chen T.-P., Su T.-L., Liang J. (2017). Plasma-activated solutions for bacteria and biofilm inactivation. Curr. Bioact. Compd..

[18] Khlyustova A., Labay C., Machala Z., Ginebra M.P., Canal C. (2019). Important parameters in plasma jets for the production of RONS in liquids for plasma medicine: A brief review. Front. Chem. Sci. Eng..

[19] Privat-Maldonado A., Schmidt A., Lin A., Weltmann K.D., Wende K., Bogaerts A., Bekeschus S. (2019). ROS from Physical Plasmas: Redox Chemistry for Biomedical Therapy. Oxidative Med. Cell. Longev..

[20] Lee Y., Ricky S., Lim T.H., Kim H., Lee E.J., Song Y., Lee S., Jang Y. (2021). An atmospheric plasma jet induces expression of wound healing genes in progressive burn wounds in a comb burn rat model: A pilot study. J. Burn Care Res..

[21] Nicol M.J., Brubaker T.R., Honish B.J., Simmons A.N., Kazemi A., Geissel M.A., Whalen C.T., Siedlecki C.A., Bilen S.G., Knecht S.D. (2020). Antibacterial effects of low-temperature plasma generated by atmospheric-pressure plasma jet are mediated by reactive oxygen species. Sci. Rep..

[22] Girard-Sahun F., Badets V., Lefrancois P., Sojic N., Clement F., Arbault S. (2019). Reactive Oxygen Species Generated by Cold Atmospheric Plasmas in Aqueous Solution: Successful Electrochemical Monitoring in Situ under a High Voltage System. Anal. Chem..

[23] Lee S., Choi J., Kim J., Jang Y., Lim T.H. (2021). Atmospheric Pressure Plasma Irradiation Facilitates Transdermal Permeability of Aniline Blue on Porcine Skin and the Cellular Permeability of Keratinocytes with the Production of Nitric Oxide. Appl. Sci..

[24] Lee S.J., Ma S.H., Hong Y.C., Choi M.C. (2018). Effects of pulsed and continuous wave discharges of underwater plasma on Escherichia coli. Sep. Purif. Technol..

[25] Hong Y.C., Park H.J., Lee B.J., Kang W.S., Uhm H.S. (2010). Plasma formation using a capillary discharge in water and its application to the sterilization of E. coli. Phys. Plasmas.

[26] Hwang Y., Jeon H., Wang G.Y., Kim H.K., Kim J.-H., Ahn D.K., Choi J.S., Jang Y. (2020). Design and Medical Effects of a Vaginal Cleaning Device Generating Plasma-Activated Water with Antimicrobial Activity on Bacterial Vaginosis. Plasma.

