# Supplementary material for: Human Trial for the Effect of Plasma-Activated Water Spray on Vaginal Cleaning in Patients with Bacterial Vaginosis
Source: Med Sci (Basel). 2022 Jun 18;10(2):33. doi: 10.3390/medsci10020033 (PMC9227462; doi:10.3390/medsci10020033)
Supplement: Supplementary file 1 [file medsci-10-00033-s001.zip › medsci-1627828-supplementary.pdf]

Supplementary Table S1. A summary on the results of STD PCR test in the betadine-treated patients.

Patient No.	Characteristics				Bacteria								PP
	Age	Height (cm)	Weight (Kg)	Hysterectomy	MH	MG	NG	UU	UP	CT	GV	TP	TV
B1	29	162.2	58.5	None	+			+			+		
B2	36	166.5	62	None				+			+		
B3	23	164.7	52.3	None						+			
B4	29	156.3	49.5	None					+				
B5	39	159.1	59.5	None	+	+		+	+	+	+		
B6	20	160.6	54	None	+				+		+		
B7	28	164.9	56.5	None					+		+		
B8	35	165.5	63.5	None							+		
B9	34	170.2	69	None					+		+		
B10	29	166.7	58.6	None						+	+		
B11	32	157.5	50.2	None					+		+		
B12	37	160.1	57.5	None				+	+		+		
B13	29	168.7	56.7	None							+		
B14	38	165.2	63.5	None							+		
B15	40	159.3	60.5	None							+		
B16	30	169	62.3	None	+				+		+		
B17	31	160.1	51.4	None					+		+		
B18	21	166.4	55.6	None	+				+		+		
B19	23	156.2	49	None					+				
B20	25	158.6	53.3	None					+		+		
B21	29	160.1	56	None				+	+		+		

B22	35	157.7	54.2	None			+	+
B23	31	163.3	60.5	None				+
B24	21	163.8	50.1	None		+		+
B25	25	156.6	52.8	None			+	+
B26	26	167.3	71.2	None			+	+
B27	29	162.1	70.5	None		+	+	+
B28	40	159.8	62.1	None			+	
B29	39	153.2	46	None			+	+
B30	31	160.3	57.5	None			+	+
B31	23	156.4	43.2	None			+	+
B32	28	160.6	53.1	None		+		+
B33	29	159.7	56	None			+	+
B34	20	161.1	49.5	None			+	+
B35	23	163.2	55.4	None		+		+
B36	28	167.1	68.5	None				+
B37	29	158.4	65.2	None	+		+	+
B38	39	161.5	63.5	None				+
B39	32	155.7	50.2	None			+	+
B40	27	162.1	55.4	None				+

PP: Protozoan parasite, MH: Mycoplasma hominis, MG: Mycoplasma genitalium, NG: Neisseria gonorrhoeae, UU: Ureaplasma urealyticum, UP: Ureaplasma parvum, CT: Chlamydia trachomatis, GV: Gardnerella vaginalis, TP: Treponema pallidum, TV: Trichomonas vaginalis

Supplementary Table S2. A summary on the results of STD PCR test in the plasma-activated water sprayed patients.

Patient No.	Characteristics				Bacteria								PP
	Age	Height (cm)	Weight (kg)	Hysterectomy	MH	MG	NG	UU	UP	CT	GV	TP	TV
P1	28	165.7	57.6	None	+			+		+	+		
P2	27	162.5	49.5	None					+		+		
P3	29	168.7	66.4	None					+				
P4	24	156.2	45	None					+				
P5	35	157.5	55.4	None					+		+		
P6	20	162	52.5	None	+			+			+		
P7	20	160.3	53	None				+	+		+		
P8	37	169.7	63.4	None							+		
P9	26	171.4	67	None	+			+			+		
P10	32	159	54.5	None					+		+		
P11	37	155.2	46.5	None					+		+		
P12	40	158.4	63	None	+			+			+		
P13	38	154.3	53.8	None				+	+				
P14	26	159.8	51.1	None				+		+	+		
P15	38	164.7	62	None							+		
P16	36	172.1	73.3	None	+			+	+	+	+		
P17	26	166.5	54.5	None					+		+		
P18	23	154.5	43.2	None					+				
P19	22	163.4	50.1	None				+			+		
P20	29	162	54.6	None	+			+	+		+		
P21	30	159.6	54.5	None	+						+		
P22	28	168.7	69	None					+		+		

P23	27	161.1	52	None					+		+		
P24	31	156.9	49.7	None				+					
P25	33	160.5	56.6	None				+	+		+		
P26	28	164.3	53.2	None					+		+		
P27	38	164.5	58.9	None				+			+		
P28	40	155.4	57.5	None					+				
P29	37	167.2	72.3	None				+					
P30	29	154.7	47.5	None					+				
P31	33	157.3	52.1	None					+		+		
P32	27	161.6	50.2	None					+		+		
P33	37	160.4	55.6	None				+		+			
P34	25	164.2	57.3	None					+		+		
P35	29	162.2	64.1	None							+		
P36	36	156.3	58.5	None					+				
P37	38	160.2	56.4	None				+					
P38	30	158.2	49	None	+				+		+		
P39	26	162.3	60.5	None	+			+			+		
P40	36	166.6	62.8	None				+					
P41	29	156.7	52.3	None					+		+		
P42	38	158.6	58.4	None							+		
P43	35	161.6	54.5	None					+				
P44	37	164.5	56.3	None				+			+		
P45	35	159.7	60.2	None				+			+		
P46	21	161.3	57.5	None	+				+		+		

PP: Protozoan parasite, MH: Mycoplasma hominis, MG: Mycoplasma genitalium, NG: Neisseria gonorrhoeae, UU: Ureaplasma urealyticum, UP: Ureaplasma parvum, CT: Chlamydia trachomatis, GV: Gardnerella vaginalis, TP: Treponema pallidum, TV: Trichomonas vaginalis
